# Optogenetic stimulation shapes dendritic trees of infragranular cortical pyramidal cells

**DOI:** 10.3389/fncel.2023.1212483

**Published:** 2023-08-01

**Authors:** Steffen Gonda, Ina Köhler, André Haase, Katrin Czubay, Andrea Räk, Christian Riedel, Petra Wahle

**Affiliations:** Developmental Neurobiology, Faculty of Biology and Biotechnology, Ruhr University Bochum, Bochum, Germany

**Keywords:** postnatal development, c-Fos, dendritic injury, dendritic retraction, rat visual cortex

## Abstract

Spontaneous or experimentally evoked activity can lead to changes in length and/or branching of neocortical pyramidal cell dendrites. For instance, an early postnatal overexpression of certain AMPA or kainate glutamate receptor subunits leads to larger amplitudes of depolarizing events driven by spontaneous activity, and this increases apical dendritic complexity. Whether stimulation frequency has a role is less clear. In this study, we report that the expression of channelrhodopsin2-eYFP was followed by a 5-day optogenetic stimulation from DIV 5–10 or 11–15 in organotypic cultures of rat visual cortex-evoked dendritic remodeling. Stimulation at 0.05 Hz, at a frequency range of spontaneous calcium oscillations known to occur in the early postnatal neocortex *in vivo* until eye opening, had no effect. Stimulation with 0.5 Hz, a frequency at which the cortex *in vivo* adopts after eye opening, unexpectedly caused shorter and somewhat less branched apical dendrites of infragranular pyramidal neurons. The outcome resembles the remodeling of corticothalamic and callosal projection neurons of layers VI and V, which in the adult have apical dendrites no longer terminating in layer I. Exposure to 2.5 Hz, a frequency not occurring naturally during the time windows, evoked dendritic damage. The results suggested that optogenetic stimulation at a biologically meaningful frequency for the selected developmental stage can influence dendrite growth, but contrary to expectation, the optogenetic stimulation decreased dendritic growth.

## Introduction

A wealth of studies has unraveled the environmental factors, signaling, and neuron-intrinsic mechanisms of dendritic growth with an emphasis on growth-promoting effects of neural activity (Wong and Ghosh, 2002; Cline and Haas, 2008). Depolarization evokes calcium currents via voltage-gated channels and NMDA receptors (Konur and Ghosh, 2005), which can trigger local protein synthesis and the release of trophic factors acting in a paracrine or autocrine manner (Wong and Ghosh, 2002; Wirth et al., 2003; Redmond, 2008). Biologically meaningful synaptic activity, for instance, elicited by rearing in an enriched environment or by training, increases dendritic complexity (Volkmar and Greenough, 1972; Greenough et al., 1979). Neurons of the upper layers respond differently than those of the deep layers which could be related to sequential genesis, migration, and arrival in the layers. Moreover, apical and basal dendrites of pyramidal cells differ functionally (Schiller et al., 2000; Milojkovic et al., 2005; Nevian et al., 2007; Major et al., 2008; Antic et al., 2010; Chalifoux and Carter, 2011) and respond differentially to morphogenetic signals. For example, antagonizing the N-methyl-D-aspartate receptor (NMDAR) subunit GluN2B in organotypic cultures (OTCs) of the rat visual cortex from DIV 7–10 results in impaired growth of basal but not apical dendrites of pyramidal cells (Gonda et al., 2020). The overexpression of the kainate receptor subunit GluK2 results in the growth of apical dendrites in layers II/III but not in layers V/VI (Jack et al., 2019). Calcium signaling evokes expression of BDNF, and neurotrophins exert layer- and type-specific effects on dendritic maturation of cortical neurons (Redmond, 2008). When BDNF is overexpressed in OTCs from the rat visual cortex until DIV 10, only the dendrites of infragranular pyramidal cells show increased growth (Wirth et al., 2003). Early postnatal overexpression of certain AMPA or kainate glutamate receptor subunits or transmembrane AMPA receptor regulatory proteins and a NETO protein auxiliary to kainate receptors increases apical dendritic complexity because the amplitude of depolarizing events driven by spontaneous activity increases with more excitatory receptors (Hamad et al., 2011, 2014; Jack et al., 2019). Stimulation frequency also plays a role. A previous study has shown that neurite growth is at its best within an optimal calcium concentration and frequency of calcium transients (Kater and Mills, 1991; Gomez and Spitzer, 1999). In this study, we tested whether dendritic complexity of channelrhodopsin2-eYFP (ChR2-eYFP) expressing cortical pyramidal neurons can be altered with repetitive optogenetic stimulation. Indeed, stimulation at a biologically meaningful range for the selected developmental stage evoked remodeling of dendrites. Contrary to expectation, we observed a reduction of dendritic length of infragranular neurons.

## Materials and methods

### Culture preparation, transfection, optogenetic stimulation, and immunostaining

**Figure 1:**
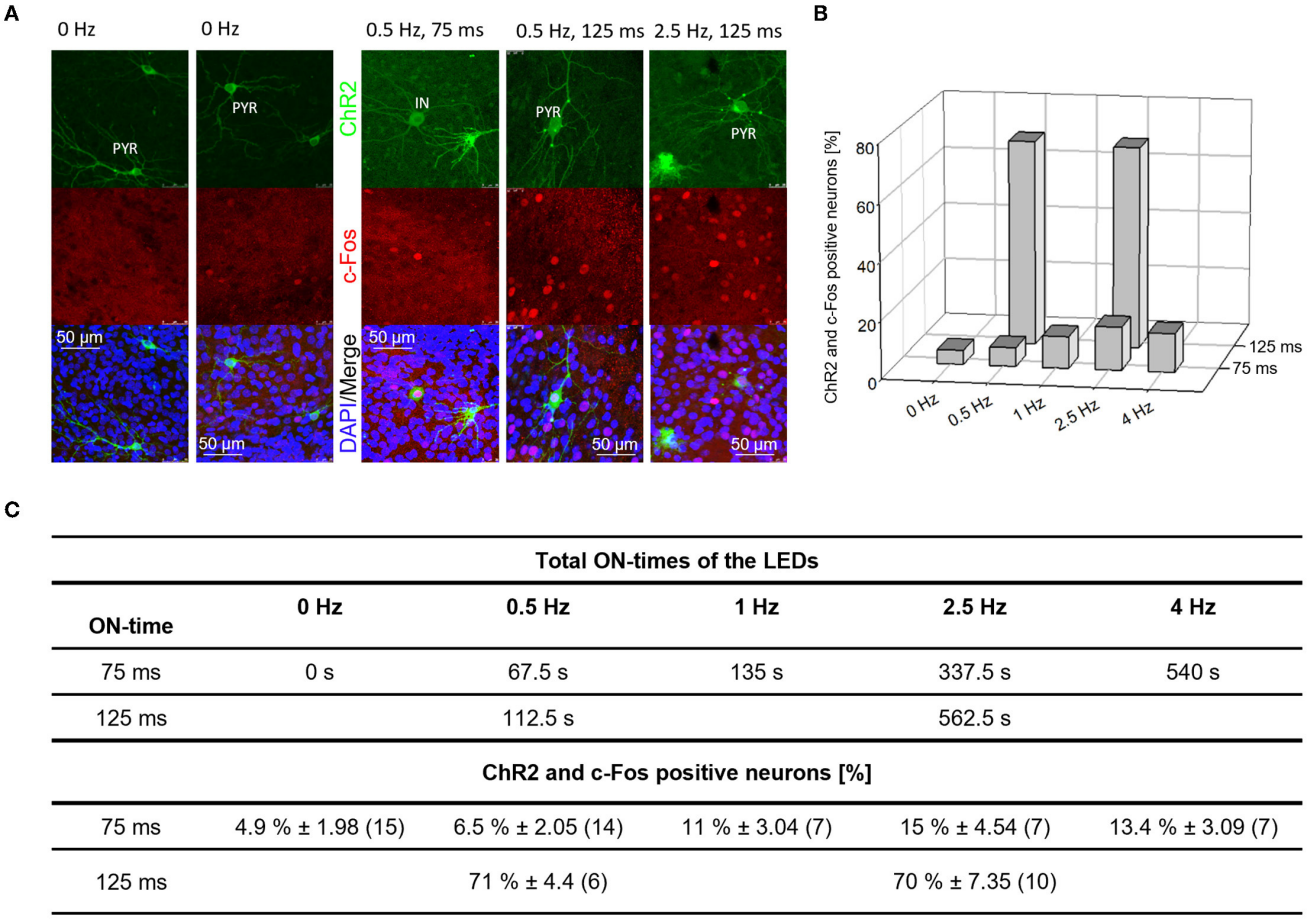
C-Fos expression in DIV 15 OTCs after optogenetic stimulation. **(A)** Photomicrographs of ChR2-eYFP/c-Fos/DAPI expressing cells in the handling control group (0 Hz) and—exemplarily—after one stimulation block (1.5 h total duration plus another 1.5 h incubation) with frequencies of 0.5 and 2.5 Hz and ON times of 75 ms and 125 ms light pulse duration. PYR, pyramidal cell; IN, Interneuron. **(B)** Percentage of ChR2-eYFP/c-Fos positive neurons vs. the frequencies and ON times tested. The longer ON time was highly effective in stimulating c-Fos protein expression in ~70% of the ChR2 transfectants. **(C)** Table with the total ON time of the LEDs (in seconds) and the proportion of ChR2-eYFP/c-Fos positive neurons from all ChR2-eYFP positive neurons (mean ± s.e.m.). The number of analyzed OTC is given in brackets; OTC from two independent preparations except for 0.5 Hz/125 ms (OTC from 1 preparation). Note the steady increase of ChR2/c-Fos double-labeled neurons from 0 to 2.5 Hz, and no further increase with 4 Hz.

Cultures were prepared from the postnatal day 1/2 pigmented rat visual cortex of five animals of both sexes/batches; slices of every animal were allocated to all conditions that were examined after the preparation. Parasagittal slices were cut at 350 μm thickness. Cultures were biolistically transfected (Helios Gene Gun, Bio-Rad, Munich, Germany) at 180 psi helium pressure (Gonda et al., 2020) with plasmids encoding CMV-driven ChR2(H134R)-eYFP (Addgene #20940) at days *in vitro* (DIV) 3 or at DIV 8. Slices were cultured in a 36°C roller incubator in a dark room to minimize light exposure. A neighboring incubator had a custom-made illumination setup equipped with a 3 x 8 individually switchable 465 nm LED (Osram Oslon SSL80; Lumitronix) controlled via an Arduino unit. OTC tubes were batchwise placed into the movable rack designed to position every OTC at 12 mm distance in front of a LED (Supplementary Figures 1A–C). The batch-internal “handling” control received the same ChR2-eYFP transfection and at the same time in the illumination setup just without any LED light (the sole variable). The handling control was essential because placing culture tubes in and out repetitively, as careful as it was done, will unavoidably generate some mechanical stress which has been shown to cause dendritic remodeling (Franze et al., 2009). Light intensity was determined with a photodiode (S130VC; Thorlabs) and a power meter (PM100D (Thorlabs) at the level of the slice cultures in the culture tubes. Light intensity was 0.7 mW/mm^2^. Cultures were exposed to daily three blocks (1.5 h each in total) of blue LED stimulation of 0.05 Hz, 0.5 Hz, and 2.5 Hz from DIV 5–10/11 and DIV 11–15 (Supplementary Figure 1D). Whole-cell illumination has been shown to efficiently evoke neuronal spiking and action potential backpropagation (Grossman et al., 2013). Approximately 3 h after one final block of stimulation at DIV 10/11 and DIV 15, cultures were fixed and immunostained with mouse anti-GFP antibody (1:1,000; clone GSN24, Sigma-Aldrich, RRID: AB_563117) as described (Gonda et al., 2020; Gasterstädt et al., 2022). To look for acute effects of the light stimulation, sets of OTC were stimulated with a range of frequencies and/or pulse duration with a single stimulation block, were returned to the roller incubator, and were fixed after 1.5 h followed by immunostaining for c-Fos (rabbit, Santa Cruz, sc-166940, 1:300; overnight), a biotinylated secondary (goat α rabbit; DAKO, #E0432, 1:750; 3–4 h) and streptavidin DyeLight594 (Thermo Fisher, #21842, 1:1,000, 2 h). Sections were briefly exposed to DAPI to label nuclei and coverslipped. The proportion of Chr2-eYFP green fluorescent neurons containing a c-Fos red fluorescent nucleus from all Chr2-eYFP neurons per OTC was determined by counting double- and single-labeled neurons in non-overlapping view fields covering the entire culture. We then plotted the percentage of double-labeled neurons per culture. The number of cultures assessed is given in Figure 1.

### Selecting stimulation frequency

For morphometry, we stimulated with light pulses flashed at 0.05, 0.5, and 2.5 Hz each with a pulse duration of 70 ms and of 140 ms. The lowest frequency matches the frequency of spontaneously occurring calcium events reported for supragranular neurons of the anesthetized mouse primary visual cortex at ~0.01 Hz at P8 and ~0.03 Hz at P11 (Rochefort et al., 2009). Calcium event frequency increases to ~0.25 Hz after eye opening and to 0.5 Hz by the end of the fourth postnatal week. The frequency of 2.5 Hz does not naturally occur spontaneously at these developmental stages (Rochefort et al., 2009). The frequency development determined in anesthetized animals reflects an overall (not single cell) activity level, and the number of contributing cells decreases substantially after eye opening (Rochefort et al., 2009). The effective frequency of individual neurons, the property we attempted to alter with the optogenetic stimulation, is presumably less than the 0.5 Hz which we identified in the present study to be of morphogenetic relevance. In an earlier study, we reported that calcium events driven by spontaneous activity via AMPA receptors are present in OTC at DIV 5 and recruit the entire network (Hamad et al., 2011). Event frequencies of approximately DIV 7–10 are ~0.01 Hz (Hamad et al., 2014; Jack et al., 2019). At DIV 11-15, event frequency determined with Oregon Bapta-1 recording has increased to 0.02–0.04 Hz, and at DIV 18–20, individual pyramidal cells expressing GCaMP6 display 0.1 Hz (Engelhardt et al., 2018; Gasterstädt et al., 2020). The overexpression of GluK2(Q), for instance, increases the frequency of calcium events to ~0.06 Hz at DIV 7–10, and this results in the growth of apical dendrites (Jack et al., 2019). Therefore, this led us to suggest that an optogenetic stimulation already at 0.05 Hz could elicit dendritic growth.

### Defining cell classes

We reconstructed completely stained pyramidal neurons with spiny dendrites and clear dendritic polarity following previously established criteria. With sparse transfections, cells ideally resided in a solitary position allowing for 3D manual reconstruction (Neurolucida, MicroBrightField, Williston, USA) done by trained observers who were blinded to condition. Neurons of supragranular layers II/III had apical dendritic tufts in layer I. Neurons of infragranular layers V/VI had apical dendrites ending in the middle layers. Neurons had a smooth main axon projecting toward the white matter, giving rise to a few obliquely ascending or horizontal collaterals which were usually thinner than the main axon and had small regular-sized boutons. We excluded horizontally oriented neurons at the uppermost and the lowermost boundary of the cultures because they may represent neurons of layer I or layer VIb/subplate. We excluded spiny apolar neurons of middle layers which resemble spiny stellates. We excluded neurons of infragranular layer V with large somata and thick- and thin-tufted dendrites in layer I because they were too rarely transfected for a meaningful analysis.

### Analysis

Reconstructions were analyzed with the Neurolucida software and the Neurolucida 360 Suite. In total, dendrites of 946 pyramidal neurons were reconstructed (at DIV 15: *n* = 134 cells @0.05 Hz; 213 cells @0.5 Hz; 145 cells @2.5 Hz; at DIV 11: *n* = 240 cells @0.05 Hz; at DIV 10: 214 cells @0.5 Hz). We determined the mean dendritic length and segment number per cell for all three stimulus frequencies. Only the 0.5 Hz stimulation altered the dendritic complexity. Because we did not see robust morphological evidence for a distinct effect of stimulus duration, we pooled the reconstructed cells of the 70 and 140 ms conditions to analyze the dendritic complexity with Sholl analyses separate for the apical dendrites and for the basal dendrites (basals of each cell pooled). For the 0.5 Hz condition at DIV 10 and DIV 15, we analyzed the following measures: maximum branch order/cell, the distance between the distalmost (apical) dendritic tip to the soma, the average length of terminal dendritic branches, the total length of the terminal dendritic branches, the average length of non-terminal (internode) dendritic segments, and the total length of the non-terminal (internode) dendritic segments.

The 2.5 Hz stimulation elicited neuronal damage. Recently, we extensively demonstrated the symptoms of dendritic injury and leaky membranes causing a halo of stained reporter protein around the somata for neurons overexpressing certain genetically encoded calcium indicators (Gasterstädt et al., 2020). The proportion of ChR2-eYFP stained neurons with dendritic beading from all ChR2-eYFP stained neurons was determined for the three stimulus conditions and the handling control at 40x magnification, using a ZEISS Axioskop microscope equipped with a discussion bridge and two trained observers who were blinded to condition. The material used for the reconstruction was also used for this assessment.

### Statistics

**Figure 2:**
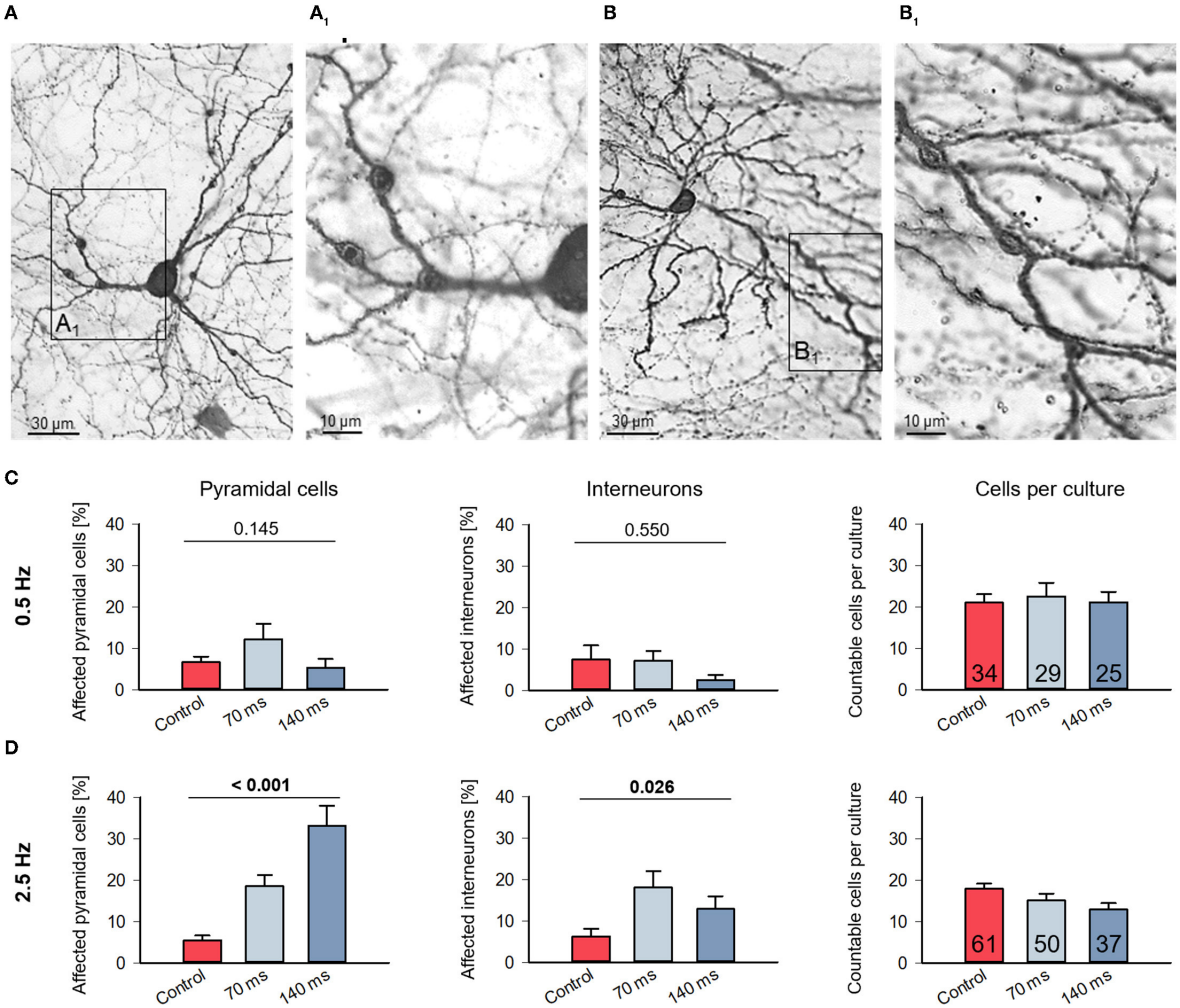
Assessment of dendritic injury after optogenetic stimulation. **(A)** Photomicrograph of a pyramidal neuron with dendritic swellings (arrowheads); **(B)** photomicrograph of an interneuron with dendritic swellings (arrowheads). The boxed areas **(A**_**1**_**)** and **(B**_**1**_**)** are shown to the right at higher magnification. Neurons which display swellings (minimum 2 each on 2 dendrites) and/or with leaky somata were scored as “affected” and expressed as percentage of all assessable neurons per OTC. **(C)** Affected cells of all layers in control and stimulated cultures from DIV 11–15 with 0.5 Hz or **(D)** with 2.5 Hz were counted. Pyramidal cells and interneurons were distinguished, which indicates that the cells assessed had a staining quality in particular of the axon sufficient for classification. The bar graphs show the percentage of affected from total cells (mean ± s.e.m.). The average number of neurons scored per culture (pyramidal and interneurons together; mean ± s.e.m) is given in the rightmost graphs. Numbers of assessed OTCs are given in the bars. *P*-values determined with Kruskal–Wallis tests (ANOVA on ranks) with Dunn's correction vs. handling control.

**Figure 3:**
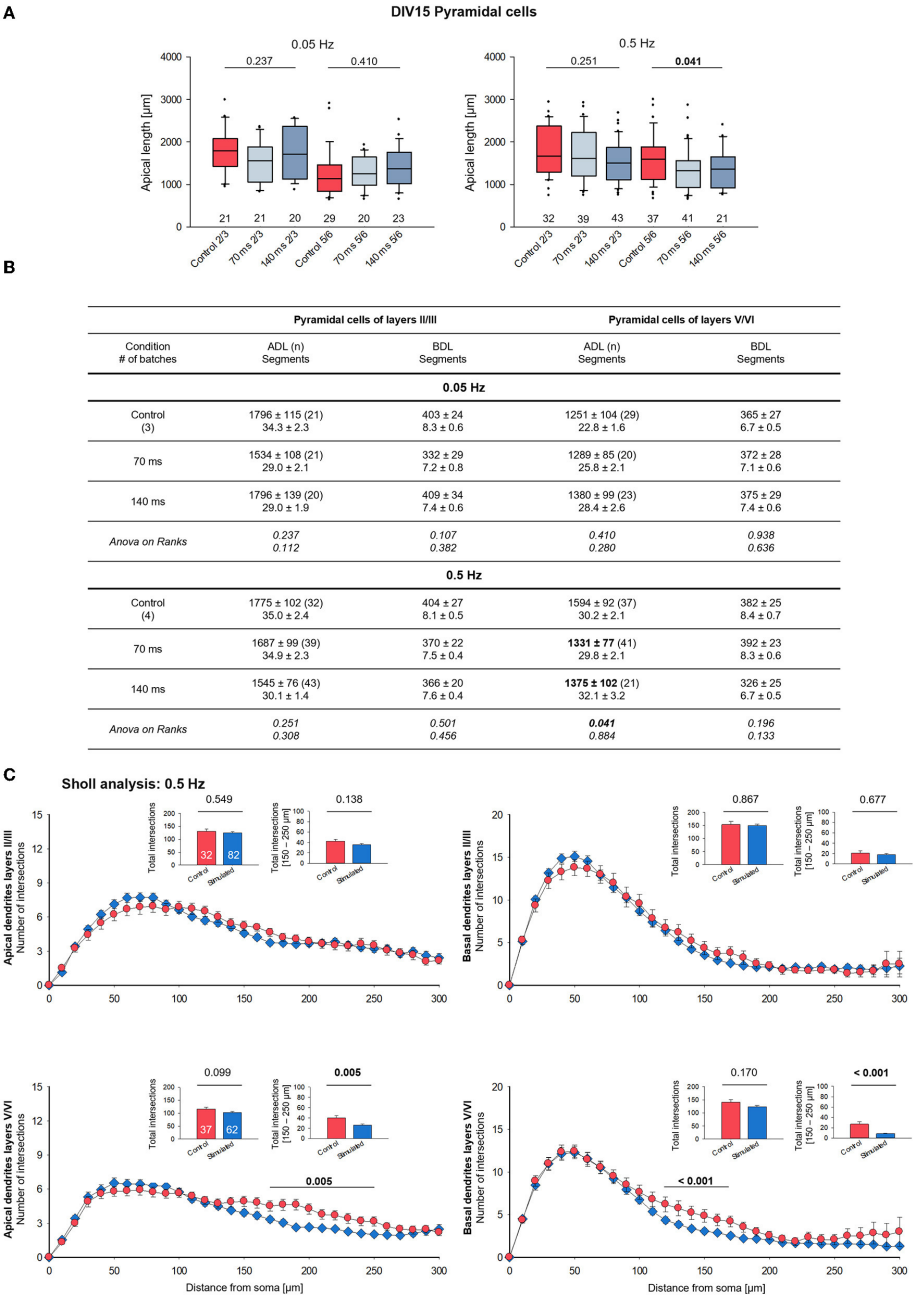
Morphometry of pyramidal cells of layers II/III and V/VI at DIV 15. **(A)** Box plots showing the length of the apical dendrites of neurons of layers II/III and V/VI for two frequencies and the two ON times. Note the reduced overall length of the apical dendrites in layers V/VI. The numbers below the boxes indicate the number of reconstructed cells. *P*-value determined with Kruskal–Wallis tests (ANOVA on ranks) vs. handling control. Handling control in red, the two ON times in shades of blue. **(B)** Table reports apical dendritic length (ADL) and basal dendritic length (BDL; average/cell), and the number of dendritic segments as mean ± s.e.m. for every set of neurons, as well as the number of cells/group and the number of independent batches. In bold, the statistically different groups of the 0.5 Hz stimulation. **(C)** Sholl analysis of apical and basal dendrites of supragranular and infragranular pyramidal cells. Seventy and one hundred forty milliseconds groups were pooled and shown in blue. The small insets (mean ± s.e.m.) report the total intersections from 0 to 300 μm distance from soma **(left)** and within selected bins **(right)**. *P*-values determined with Mann–Whitney rank-sum tests are given above the bars.

**Figure 4:**
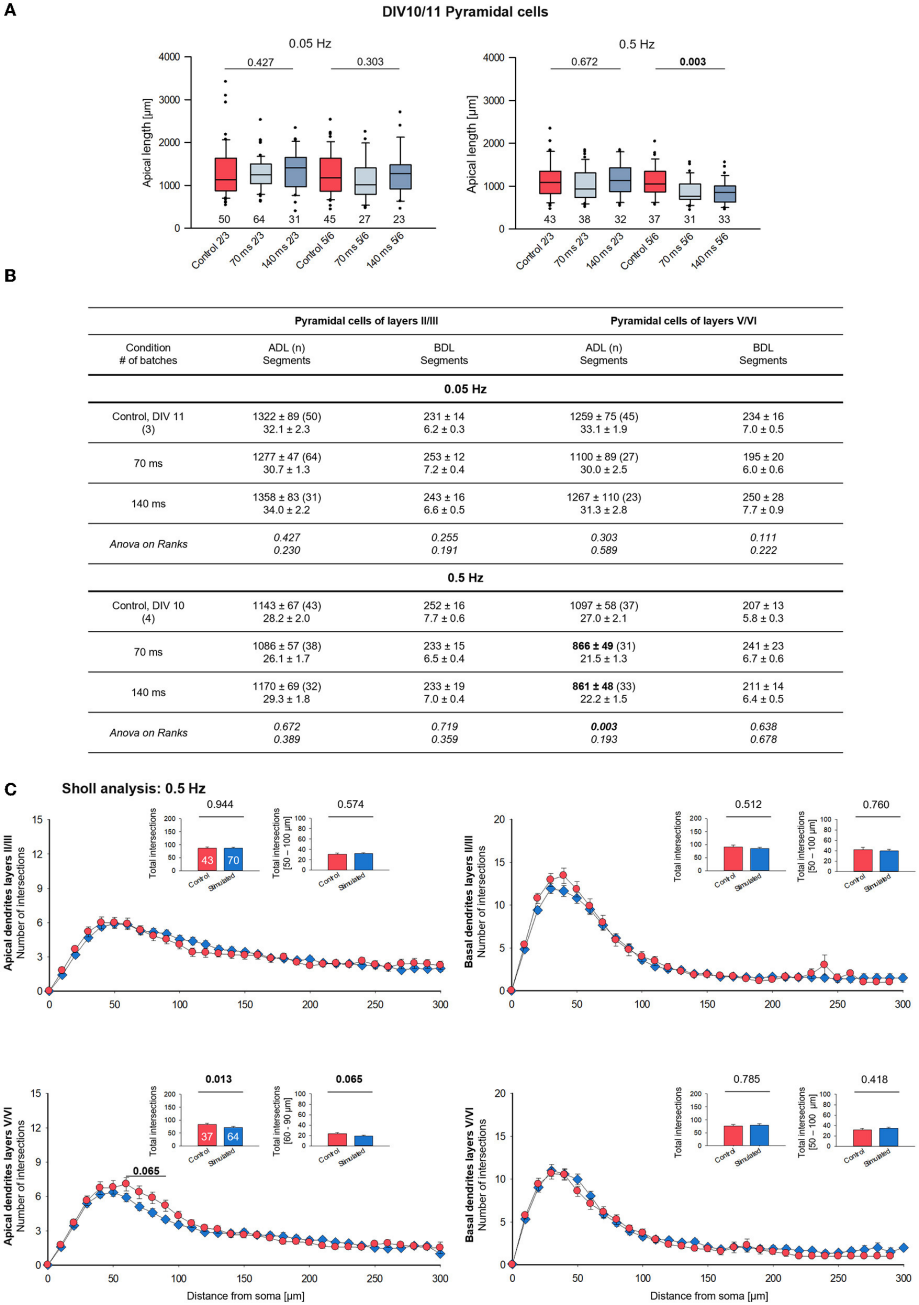
Morphometry of pyramidal cells of layers II/III and V/VI at DIV 10/11. **(A)** Box plots showing the length of the apical dendrites of neurons of layers II/III and V/VI for two frequencies and the two ON times. Note the reduced overall length of the apical dendrites in layers V/VI with the 0.5 Hz stimulation. The numbers below the boxes indicate the number of reconstructed cells. *P*-value determined with Kruskal–Wallis tests (ANOVA on ranks) vs. handling control. Handling control in red, the two ON times in shades of blue. **(B)** Table reports apical dendritic length (ADL) and basal dendritic length (BDL; average/cell), and the number of dendritic segments as mean ± s.e.m. for every set of neurons, as well as the number of cells/group and the number of independent batches. In bold, the statistically different groups of the 0.5 Hz stimulation. **(C)** Sholl analysis of apical and basal dendrites of supragranular and infragranular pyramidal cells. Seventy and one hundred forty milliseconds groups were pooled and shown in blue. The small insets (mean ± s.e.m.) report the total intersections from 0 to 300 μm distance from soma **(left)** and within selected bins **(right)**. *P*-values determined with Mann–Whitney rank-sum tests are given above the bars.

**Figure 5:**
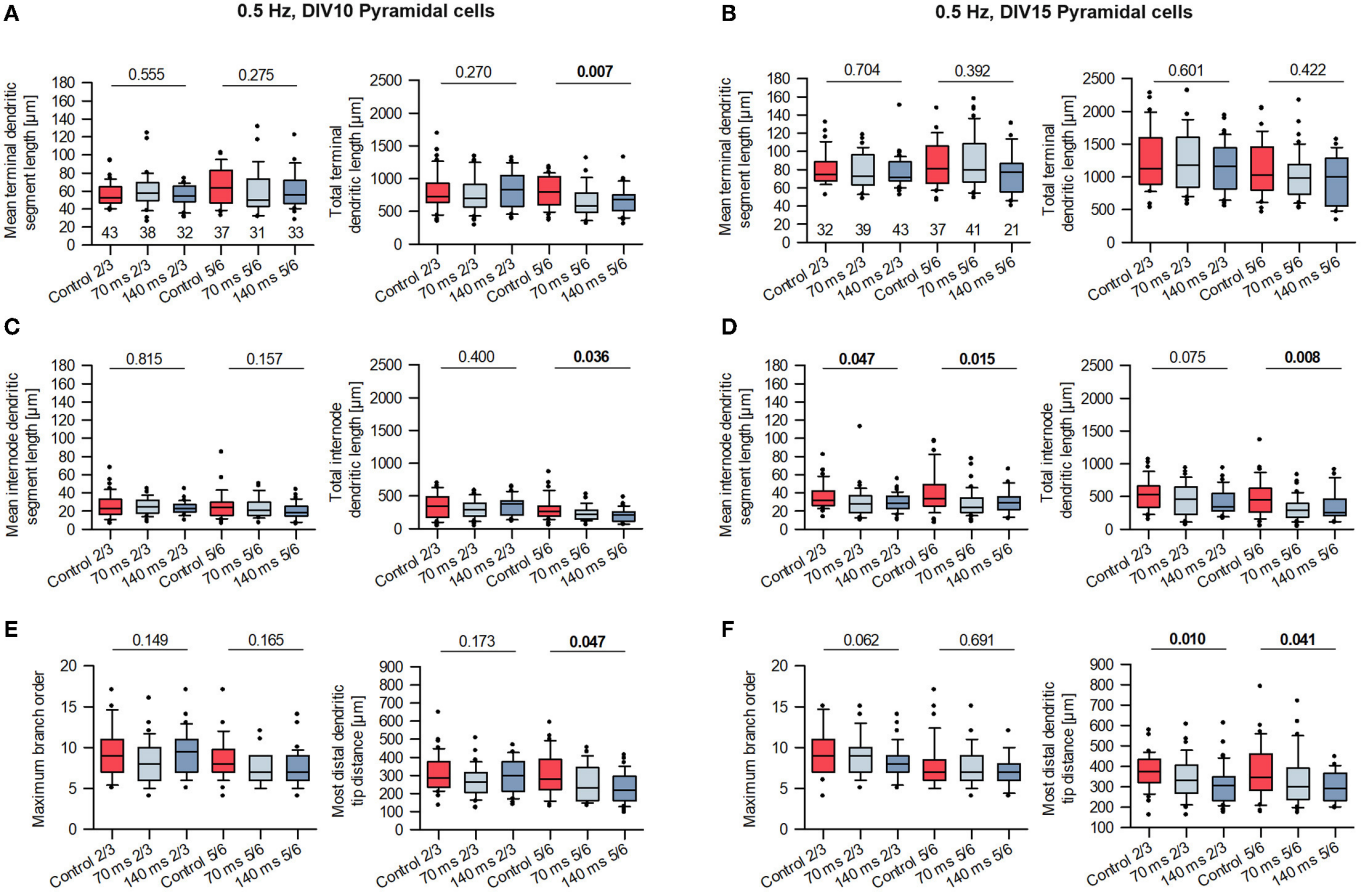
Morphometry of 0.5 Hz stimulated pyramidal cells of layers II/III and V/VI at DIV 10 and 15. **(A)** Mean terminal dendritic segment length **(left)** and total terminal dendritic segment length **(right)** at DIV 10. **(B)** Mean terminal dendritic segment length **(left)** and total terminal dendritic segment length **(right)** at DIV 15. **(C)** Mean internode dendritic segment length **(left)** and total internode dendritic segment length **(right)** at DIV 10. **(D)** Mean internode dendritic segment length **(left)** and total internode dendritic segment length **(right)** at DIV 15. **(E)** Maximum branch order **(left)** and distance from soma to distalmost dendritic tip **(right)** at DIV 10. **(F)** Maximum branch order **(left)** and distance from soma to distalmost dendritic tip **(right)** at DIV 15. Handling control in red, the two ON times in shades of blue. The numbers below the boxes in **(A, B)** indicate the number of reconstructed cells. *P*-value determined with Kruskal–Wallis tests (ANOVA on ranks) vs. handling control, significant differences in bold face.

Reconstructions were derived from 20 independent preparations (DIV 10: 51 OTC/4 batches @0.5 HZ; DIV 11: 29 OTC/3 batches @0.05 Hz; DIV 15: 33 OTC/3 batches @0.05 Hz; 62 OTC/4 batches @0.5 Hz; 48 OTC/6 batches @2.5 Hz). Statistical comparison was performed vs. the handling controls using non-parametric Mann–Whitney rank-sum tests or Kruskal–Wallis ANOVA on ranks with Dunn's correction for multiple testing where appropriate. The number of cells was analyzed, and the *p*-values are given in Figures 2–**5** and Supplementary Figure 3.

## Results

Organotypic cortex cultures preserve the cortical architecture and develop spontaneous action potential activity and activity-dependent neurotrophin expression (Gorba et al., 1999; Klostermann and Wahle, 1999; Chattopadhyaya et al., 2004; Baho and Di Cristo, 2012). Our hypothesis was that we could evoke dendritic growth with an optogenetic stimulation at frequencies that are within the range of those that occur naturally during or shortly after the selected developmental time window.

### Optogenetic stimulation evokes c-fos expression

Although the action of channel rhodopsin for neuronal depolarization and spiking is almost textbook knowledge, we tested first whether the stimulation evokes activity in the early postnatal OTC using the activity-dependent expression of c-Fos protein as read-out. The immediate early gene c-Fos becomes expressed and nuclear enriched upon changes in the electrical activity. One block of stimulation was done (duration 1.5 h) followed by 1.5 h in the roller incubator. Representative photomicrographs (Figure 1A) and the quantification (Figure 1B) show that very few ChR2-eYPF neurons expressed c-Fos in the handling control condition. The 0.5 Hz stimulation with 75 ms pulse duration yielded a marginal increase of double-labeled neurons which further increased at 1 and 2.5 and 4 Hz. Substantially higher percentages were seen with 125 ms pulse duration at 0.5 Hz and, similarly, with 2.5 Hz (Figures 1B, C). Furthermore, the overall level of c-Fos positive nuclei was increasing with frequency and pulse duration in the OTC suggestive of an induction of network activity via action potentials generated by the ChR2-positive neurons. Eventually, we decided to run the dendrite analysis with two pulse durations, 70 and 140 ms (varying by a factor of 2), and with three frequencies (varying by a factor of 10 and 5, respectively). The frequency of 0.05 Hz is within the range of spontaneously occurring calcium events at the early time window *in vivo* (Rochefort et al., 2009) and in OTC (Hamad et al., 2014; Jack et al., 2019). The frequency of 0.5 Hz reflects the “next higher” frequency level (Rochefort et al., 2009).

### Optogenetic stimulation can evoke neuronal damage

We already noted dendritic swellings in some ChR2-eYPF fluorescent neurons of the 2.5 Hz condition suggestive of neuronal damage (Figure 2). The symptoms of dendritic injury, from mild to severe to cell death, have been extensively documented recently for neurons overexpressing genetically encoded calcium indicator proteins (Gasterstädt et al., 2020) and with live imaging in neurons overexpressing AMPA receptor subunits treated with the receptor ligands (Hamad et al., 2011; Jack et al., 2019; Gonda et al., 2020). We therefore assessed the DIV 11–15 light-stimulated, ChR2+ immunoperoxidase-stained neurons. Numbers obtained for this type of analysis were naturally higher than the number of reconstructed cells because symptoms of neurite injury can also be easily detected in less completely stained neurons which we did not consider for reconstruction. Pyramidal cells with spiny dendrites (Figures 2A, A_1_) and interneurons with smooth or sparsely spinous dendrites (Figures 2B, B_1_) displayed swellings along the dendrites; they were mildly affected and still intact. Quantitatively, at 0.5 Hz stimulation, the percentages of affected neurons were at 5–10% and within the range seen also in the handling control (Figure 2C). On average, approximately 20 sufficiently well-stained neurons were assessable in every OTC of the three conditions. In contrast, at 2.5 Hz stimulation, the percentage of affected neurons was significantly higher. Pyramidal cells appeared more severely affected than interneurons, and overall, the number of assessable neurons per light-stimulated OTC tended to decrease suggesting that cell death has taken a certain toll already during the stimulation period (Figure 2D). This suggested that a biologically relevant frequency does not affect neuronal survival, whereas the higher frequency at this developmental stage damages pyramidal cells and interneurons.

### Optogenetic stimulation can decrease dendritic complexity

Representative examples of completely stained supragranular and infragranular pyramidal cells of the 0.5 Hz condition are shown as photomicrographs (Supplementary Figure 2A) and skeletal drawings (Supplementary Figure 2B). Quantitatively, the 0.05 Hz/70 ms and 0.05 Hz/140 ms stimulation had no effect on apical dendritic complexity in both supragranular and infragranular pyramidal cells (Figure 3A, left; Figure 3B). In contrast, compared to the handling control, the 0.5 Hz stimulation at both pulse durations significantly reduced apical dendritic length, but not the number of segments, of infragranular pyramidal cells (Figure 3A, right; Figure 3B). The averages of basal dendritic length and branch number were not affected (Figure 3B). Apical length and branching of neurons of layers II/III were not significantly different from control. Since we could not find consistent differential effects of the pulse duration, we pooled the neurons of the 70 and 140 ms condition for the Sholl analysis of the 0.5 Hz stimulated cells. Apical dendrites of neurons of layers II/III were not affected, and the Sholl curves of control and stimulated neurons were overlapping (Figure 3C). A highly significant shift to less apical complexity emerged at 150–250 μm distance from the soma of layer V/VI pyramidal cells; moreover, also basal dendrites of layer V/VI pyramidal cells were less complex than those of the control cells (Figure 3C).

The 2.5 Hz stimulation had no effect on apical dendritic complexity (Supplementary Figure 3A). Basal dendritic length and branching tended to be lower but did not reach significance. Basal branching was undershooting the control cells in the Sholl analysis (Supplementary Figure 3B) in two bins proximal to the soma, without deviating at larger distances to the soma. For the 2.5 Hz condition, care was taken to reconstruct overall healthy neurons and not cells with fragmented dendrites. Due to the overall lower number of healthy transfectants, a total of six batches had to be exploited to obtain cell numbers comparable to those sampled for the other frequencies. This might have led to somewhat higher interbatch variability.

The lack of effect of the 0.05 Hz stimulation at DIV 15 prompted the question toward a response in younger neurons. Stimulation with 0.05 Hz from DIV 5–11 did not evoke any differences in apical and basal length and branching compared to the handling control cells (Figure 4A, left; Figure 4B). However, stimulation with 0.5 Hz from DIV 5–10 evoked significantly shorter apical dendrites of layer V/VI pyramidal neurons (Figure 4A, right; Figure 4B). Apical branching was not impaired, and basal dendrite length and branching remained at the control level. The Sholl curve of stimulated DIV 10 neurons tended to undershoot the control curve between 50 and 100 μm from the soma (Figure 4C).

A more refined analysis of the stimulated cells revealed a reduction of the total but not the mean length of the terminal dendritic branches of layer V/VI pyramidal cells at DIV 10 (Figure 5A). This is suggestive of a pruning of distal dendritic elements compared to control cells and to supragranular pyramidal cells. The difference was no longer present at DIV 15 (Figure 5B); now, both measures were substantially longer and much more variable compared to DIV 10. Furthermore, the total, but not the mean length of non-terminal (internode) dendritic segments of layer V/VI pyramidal cells was reduced at DIV 10 (Figure 5C); in addition, total and mean internode length was reduced at DIV 15 (Figure 5D). Both measures were overall longer than at DIV 10 indicative of ongoing growth of control and stimulated neurons. Interestingly, a subtle reduction was seen at DIV 15 for layer II/III pyramidal neurons indicating that this subset is also, albeit mildly, affected by the stimulation. Interestingly, the maximum branch order at DIV 10 (Figure 5E, left) was overall similar to that at DIV 15 (Figure 5F, left), indicating that the dendritic pattern is present at DIV 10. The distance between the distalmost (apical) dendritic tip to the soma of supragranular and infragranular pyramidal cells was reduced at DIV 10 (Figure 5E, right) and at DIV 15 (Figure 5F, right), suggesting a more stunted growth for both subsets of pyramidal cells. Apparently, the 0.5 Hz stimulated neurons form apical dendritic branches more rapidly resulting in a more compact tree, but overall, they do not form excessive numbers of dendritic branches. Together, the results suggest that optogenetic stimulation at a biologically meaningful frequency can elicit dendritic remodeling. The reduction of terminal segment length at DIV 10 could be interpreted as a regressive event as pruning of apical dendrites of infragranular pyramidal neurons. The shorter internode segments and maximum height at DIV 10 and DIV 15, however, rather argue for a growth delay.

## Discussion

The optogenetic stimulation elicited activity of the ChR2-expressing neurons as indicated by c-Fos protein expression and ChR2/c-Fos double-labeled neurons increased with increasing pulse frequency and pulse duration. One might ask why not all ChR2 neurons turned c-Fos positive. With the timeline used, we were near the half-life of c-Fos expression. Thus, apart from the technical aspect of antibody penetration in slice cultures, the expression might have already declined to below the detection level in some ChR2-positive neurons. It suggested that an acute effect could have yielded even higher numbers of c-Fos positive neurons. Furthermore, stimulation frequencies close to the range of naturally occurring frequencies can alter dendritic complexity. Against hypothesis, however, the 0.5 Hz stimulation reduced dendritic complexity selectively of infragranular pyramidal cells in both time windows.

Initially, during development, the network grows extensively, followed by a period of decreased growth and pruning of weak connections. Pruning of dendrites is important for the refinement of the network (Neely and Nicholls, 1995). The expression of constitutively active CaMKII in Xenopus optic tectum neurons restricts the growth of dendrites, whereas the inhibition of CaMKII enhances dendritic growth (Wu and Cline, 1998). The activation of cytosolic and membrane-associated CamKI is the essential first step of NMDA receptor and calcium influx-dependent dendritic elongation and branching of hippocampal neurons, involving MAP kinase signaling and CREB activation further downstream (Wayman et al., 2006). The growth of neurites follows a hormetic principle, in that factors which promote dendritic growth can also evoke the opposite effect when administered at too high doses (Kater and Mills, 1991; Gomez and Spitzer, 1999; Rubio-Casillas and Fernández-Guasti, 2016). For example, in pyramidal cells, GluN2B receptor signaling promotes basal dendritic growth (Gonda et al., 2020), but excessive NMDA receptor activation, e.g., by disinhibition, results in dendritic injury and retraction (Ikegaya et al., 2001; Nishimura et al., 2008; Martin and Wellman, 2011), and neurons which survive such excitotoxic activation show a decreased dendrite growth (Monnerie et al., 2003). Nevertheless, neurons are capable of preventing cell damage by homeostatically downregulating transmitter receptors which renders neurons less excitable (Wang et al., 2016) and subsequently less responsive to growth-promoting stimuli. Calcium is a key player in dendritic growth and maintenance (Wayman et al., 2006). The disruption of calcium signaling, e.g., upon expression of the indicator GCaMP3, leads to less complex apical dendrites (Gasterstädt et al., 2020) because calcium indicator proteins are sequestering calcium. For instance, chronic optogenetic stimulation of DIV 21–30 pyramidal cell populations in sparsely transfected Stoppini-type cultures of the mouse somatosensory cortex has been shown to reduce the frequency and amplitude of UP states of the transfectants via homeostatic mechanisms to reduce their excitability and to cause a synaptic decoupling between ChR2-expressing stimulated and neighboring ChR2-negative neurons (Liu et al., 2023). Assuming that such a functional shift might have happened already in our much younger cultures, such a cell-intrinsic effect could evoke the reduction of apical dendritic length of supra- and infragranular pyramidal cells.

Furthermore, inhibition may play a role. For instance, chemogenetic silencing of neurons reduces the growth of apical dendrites of layer II/III neurons and their axonal collateralization (Gasterstädt et al., 2022). In line, excessive hyperpolarization by axosomatic parvalbumin-positive interneurons has been implicated in the stunted dendritic growth typical for Rett syndrome (Armstrong et al., 1995; Durand et al., 2012; Tomassy et al., 2014; Patrizi et al., 2020). In Down's syndrome, overinhibition and a reduced BDNF expression have been implicated in the reduction of dendritic complexity (Dierssen et al., 2003; Zorrilla de San Martin et al., 2018). Strong inhibition weakens action potential backpropagation into dendrites, and this could reduce release of neurotrophins. Backpropagation is known to trigger BDNF release (Kuczewski et al., 2008) which can increase dendritic complexity (Wirth et al., 2003; Redmond, 2008). Overexpressing BDNF in P25 ferret visual cortex slice cultures evokes a massive sprouting of basal dendrites via autocrine signaling; however, these short dendrites and in particular their spines are highly unstable. In addition, dendritic branches of ~90 μm from the soma are reduced, indicating that excessive BDNF destabilizes dendritic growth (Horch et al., 1999). We could not see such an increase in the number of basal dendrites nor any significant reduction of higher order basal branches (except for in the 2.5 Hz experiment, possibly). Rather, between DIV 10/11 and DIV 15, the basal dendrites of supra- and infragranular pyramidal neurons grow substantially. We, therefore, suggest that the optogenetic stimulation did not elicit excessive BDNF production in the transfectants, and furthermore, the stimulation *per se* did not have any global negative effect on the neuronal structure.

Transcranial optogenetic stimulation (180 light pulses, each 3 s long with 7 s intervals) to activate ChR2-positive medial prefrontal cortex L2/3 pyramidal cells in mice from P7-12 elicits an increase of dendritic length and an increase in the power of endogenous oscillations (Bitzenhofer et al., 2021). The length increase is transient and normalizes to control levels at P23 and older (Bitzenhofer et al., 2021). Although postnatal age is comparable to our developmental time windows, we could not observe any length increase. Probably, in addition to the context (e.g., species, regional, and cell-type specific ion conductances, *in vivo*/*ex vivo*), the exact stimulation protocols are of critical importance. Bitzenhofer et al. (2021) also reported an increase in inhibition with more parvalbumin-positive neurons in the stimulated cortex which have longer dendrites and exert stronger inhibition as suggested by slightly larger amplitudes of inhibitory postsynaptic potentials. The optogenetic activation of pyramidal neurons in DIV 19 hippocampal slice cultures increases the number and the size of gephyrin-positive postsynaptic inhibitory domains along their proximal apical dendrites within 24 h in an NMDA receptor-dependent manner (Flores et al., 2015), and GABAergic terminals grow larger and release more GABA upon NMDA receptor stimulation (Fiszman et al., 2005). Alternatively, the optogenetic activation of layer II/III pyramidal cells could have contributions. For instance, in the somatosensory cortex, descending layer II/III projections suppress the spontaneous activity of layer V pyramidal cells via the activation of dendrite-targeting interneurons (Pluta et al., 2019). This could explain why our infragranular pyramidal cells were so strongly affected. Together, it remains to be shown if an engagement of inhibitory neurons via network activity elicited by the 0.5 Hz stimulation might have contributed to the substantial apical dendritic length reduction of infragranular pyramidal cells.

Repetitive ChR2-evoked depolarization might have compromised metabolism and energy allocated to neuronal compartments. Bioenergetics might deliver a simple reason: If neurons are substantially depolarized, they may suffer energy shortage and start to save on neurite outgrowth. Action potential burst activity reduces dendritic ATP levels and cortical dendrites are more vulnerable to energetic and redox fluctuations than somata (Hasel et al., 2015). Indeed, healthy mitochondria that are actively transported into dendrites are essential for dendrite growth and maintenance (Tsubouchi et al., 2009; Fukumitsu et al., 2015; Shen et al., 2019). Though worth a thought, the metabolic argument falls short of explaining why supragranular pyramidal neurons were only subtly affected. Moreover, one would expect to see an even stronger growth impairment after the 2.5 Hz stimulation. ChR2-mediated sodium currents render the neurons more active, and it has been shown in zebrafish spinal cord that electrically highly active neurons have the lowest dendritic filopodial dynamics (Kishore and Fetcho, 2013) and have high chances to survive (Warm et al., 2022). In the rodent retina, depolarization-evoked release of calcium from intracellular stores stabilizes dendrites (Lohmann et al., 2002). Over time, this may result in less complex dendritic trees than control neurons. In this view, the ChR2 stimulation would not trigger a regressive process but would rather stabilize the dendritic dimensions present at the beginning of the stimulation. Indeed, the total internode dendritic length present at DIV 15 (see Figure 5D, right) is close to the measure obtained at DIV 10 (see Figure 5C, right). For axons, excessive calcium influx evoked by ChR2 stimulation into growth cones stops axonal growth (Huang et al., 2017). In line, stimulating with 4 and 10 Hz, but not 0.1 and 1 Hz, has been shown to temporarily slow down axonal elongation (Malyshevskaya et al., 2013), suggesting that the higher frequencies resemble the spontaneous activity level at a later time period when axonal elongation has ceased, and axons instead elaborate terminal branches.

These arguments do not explain why the infragranular neurons were strongly affected by the 0.5 Hz stimulation, whereas supragranular neurons were only mildly affected. However, the study (Malyshevskaya et al., 2013) lends support to our interpretation that “more of the same” will not result in morphological changes. Indeed, our 0.05 Hz stimulation was morphogenetically ineffective at DIV 10 and at DIV 15. It seems as if morphological changes can be elicited with activity patterns which resemble but do not far exceed those adopted at “the next developmental level.”

The 2.5 Hz stimulation did not alter apical dendritic complexity and also did not evoke c-Fos expression higher than the proportion reached with 0.5 Hz stimulation, suggesting a saturation. Tentatively, the small differences toward less branched basal dendrites occurring in a few Sholl bins have been caused by interbatch variability since six independent preparations had to be exploited for this condition to obtain sufficient numbers of neurons. Furthermore, the higher incidence of neuronal damage of 2.5 Hz stimulated pyramidal cells asks for caution with interpretation. For survival, neurons depend on optimal activation levels via spontaneous activity (Warm et al., 2022). The suppression of spontaneous activity and synchronized burst discharges in organotypic neocortical slices of neonatal mice by application of TTX increases the apoptosis rate (Heck et al., 2008). Non-physiological activity patterns evoked by optogenetic stimulation in primary hippocampal cultures can also increase the cell death rate (Wong Fong Sang et al., 2021). Apparently, the 2.5 Hz stimulation with higher activity levels of the ChR2 transfectants and presumably also of numerous non-transfected, c-Fos positive neighboring neurons of the network is beyond of what the still immature DIV 11–15 neurons can cope with.

A biologically intriguing explanation of the layer-specific effect comes from looking at what happens in developing the visual cortex *in vivo* during the time window. Immature postmigratory pyramidal cells initially have their apical dendrites ending in layer I. Perinatally, a period of remodeling begins. Corticothalamic layer VI pyramidal cells retract their apical dendrites which now terminate in layer IV. Between P7 and P10 in the visual cortex, numerous pyramidal cells of layer V forming callosal projections as indicated by retrograde labeling retract the apical dendrites which thereafter end rather unconspicously in middle layers (Koester and O'Leary, 1992; Kasper et al., 1994). Concurrently, the large subcortically projecting layer V pyramidal neurons elaborate their apical tufts in layer I. This fits well with the apical length reduction we observed with the 0.5 Hz stimulation from DIV 5–10. Indeed, the total terminal length of infragranular pyramidal cells was reduced at DIV 10 but no longer at DIV 15, which could be interpreted as a pruning of distal dendritic elements. Regressive events are influenced by neuronal activity. Indeed, in the prefrontal cortex, cholinergic signaling via α5 nicotinic receptors has been implicated in the remodeling of layer VI apical dendrites (Bailey et al., 2012). The role of activity has been extensively demonstrated for spiny stellates of layer IV which remodel their initially polarized pyramidal-like appearance. The apical dendrite regresses, and the basal dendrites elaborate to form the typical stellate pattern with dendrites restricted to single barrels in layer IV, which at the same time becomes heavily innervated by thalamocortical afferents (Lund et al., 1991; Callaway and Borrell, 2011; Nakazawa et al., 2018). However, when deprived of thalamocortical input activity, spiny stellates of the mouse somatosensory cortex remain polarized and do not adopt radial basal dendritic fields (Li et al., 2013). The GluN2B receptor regulates this patterning in a cell-autonomous manner, and a lack of GluN2B results in stellate cells extending dendrites into multiple barrels (Espinosa et al., 2009). In OTC, the driving force of subcortical afferents is lacking, and so, dendritic remodeling may proceed more slowly in OTC. Suggestively then, the optogenetic stimulation accelerates the naturally occurring remodeling, and this way helps infragranular neurons to acquire their characteristic morphology. Although intriguing, infragranular pyramidal cells were not the only ones impaired by the 0.5 Hz stimulation.

As shown by our staining, ChR2-YPF labeled the neuronal membrane completely. Activation of ChR2 evokes a sodium influx which may subsequently recruit calcium signaling. An evoked depolarization and calcium influx is considered beneficial for plastic changes when it happens synaptically. In contrast, extrasynaptic signaling can be neurotoxic due to a shut-off of the CREB pathway and BDNF production (Hardingham et al., 2002). In hippocampal neurons, the shut-off emerges between DIV 7 and DIV 12, and at DIV 12, extrasynaptic NMDA receptor activation overrides the beneficial CREB-activating signal elicited by synaptic NMDA receptors (Hardingham and Bading, 2002). In the neocortex, the ontogenetically older pyramidal cells of layers V/VI are more mature than the supragranular pyramidal cells and possibly switch to CREB shut-off earlier during development. This assumption could explain why infragranular pyramidal neurons were strongly affected by optogenetic stimulation. In summary, our results suggest that optogenetic stimulation at a biologically meaningful frequency for the selected developmental stage can influence dendrite remodeling. Contrary to expectation, the stimulation decreased dendritic growth in a layer-specific manner. The underlying mechanisms remain to be elucidated.

## Data availability statement

The original contributions presented in the study are included in the article/Supplementary material, further inquiries can be directed to the corresponding author.

## Ethics statement

The animal study was reviewed and approved by Ruhr University Bochum Animal Research Board.

## Author contributions

PW conceived the experiments. AR prepared the cultures. SG, IK, AH, KC, CR, and PW performed the experiments, reconstructions, and data management. SG, IK, and PW analyzed the results. PW wrote the manuscript. All authors contributed to the article and approved the submitted version.
