# Supplementary material for: Optogenetic stimulation shapes dendritic trees of infragranular cortical pyramidal cells
Source: Front Cell Neurosci. 2023 Aug 1;17:1212483. doi: 10.3389/fncel.2023.1212483 (PMC10427221; doi:10.3389/fncel.2023.1212483)
Supplement: Supplementary file 1 [file Data_Sheet_1.pdf]

Supplementary Information

Optogenetic stimulation shapes dendritic trees of infragranular cortical pyramidal cells

Steffen Gonda[1 +], Ina Köhler[1 +], André Haase[1], Katrin Czubay[1], Andrea Räk[1], Christian Riedel[1], Petra Wahle[1*]

[1] Developmental Neurobiology, Faculty of Biology and Biotechnology, Ruhr University Bochum, 44801 Bochum, Germany

+ shared first authors, * correspondence

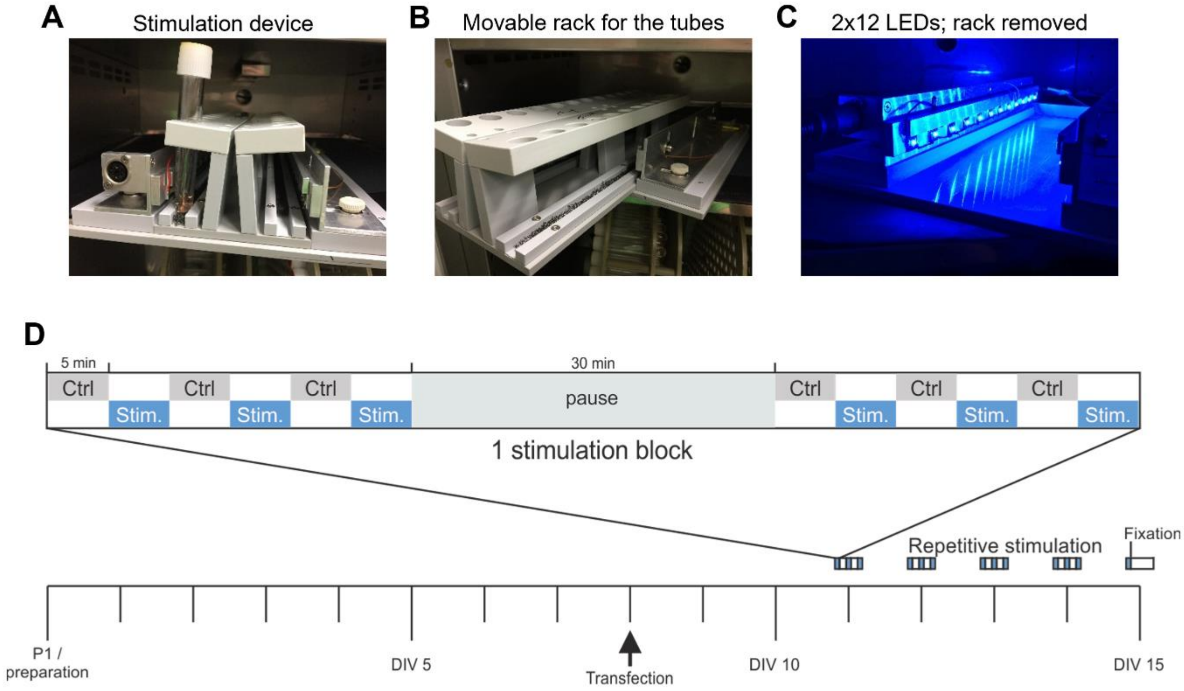

Supplementary Fig. S1. Stimulation set-up and stimulation protocol.

*(**A**) Custom-built stimulation device. **(B)** Movable rack to place the culture tubes in exact distance to each LED. The bottom groove was cushioned with a strip of black spongy plastic to reduce mechanical shock. **(C)** LED band on one side. (**D**) Experimental timeline with blocks of optogenetic stimulation, 3/day in early morning, mid-day and late afternoon starting at DIV 11. For the younger time window transfection was at DIV 3, and stimulation was from DIV 5-10/11. About 3 h after a final stimulation block at DIV 10/11 and DIV 15, respectively, cultures were fixed and stained.*

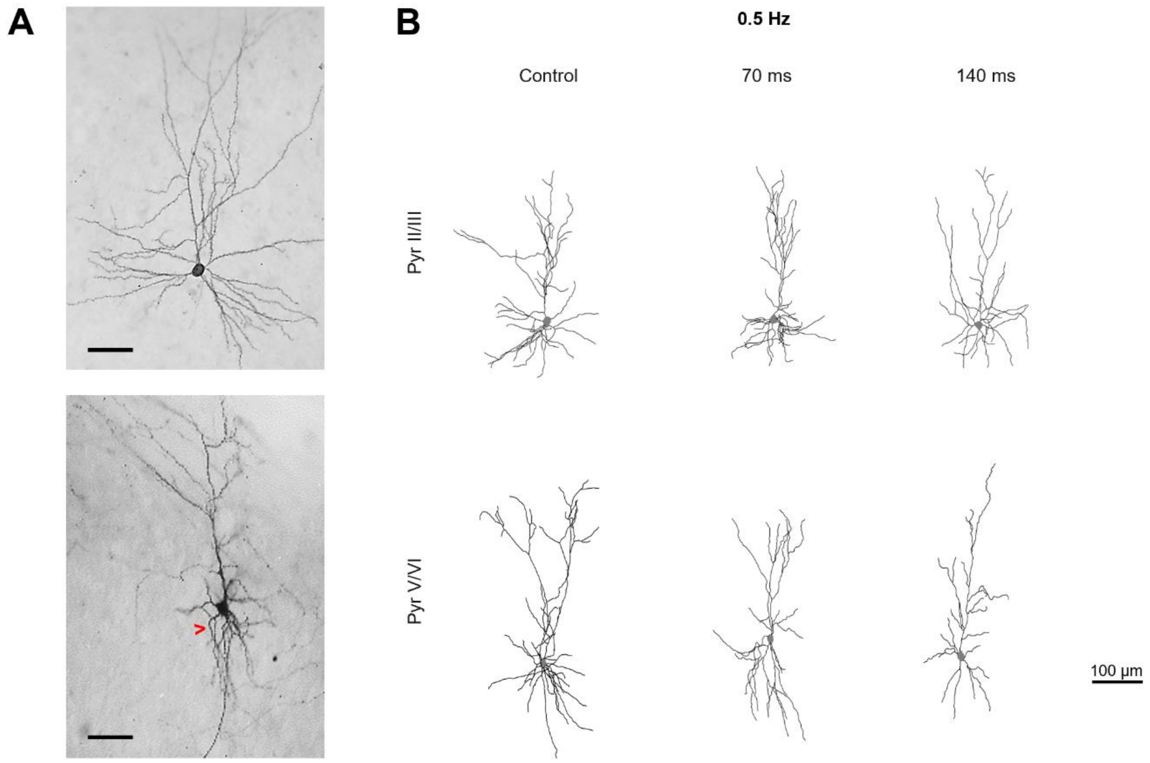

Supplementary Fig. S2. Morphology of pyramidal cells of layers II/III and V/VI.

(A) *photomicrographs of completely stained pyramidal cells. Red arrow marks axon. Scale bar: 50 µm.*
(B) *Skeletal drawings of representative neurons of the 0.5 Hz condition.*

A

DIV15 Pyramidal cells

Condition<br># of batches	Pyramidal cells of layers II/III: ADL (n)<br>Segments	Pyramidal cells of layers II/III: BDL<br>Segments	Pyramidal cells of layers V/VI: ADL (n)<br>Segments	Pyramidal cells of layers V/VI: BDL<br>Segments
**2.5 Hz**				
Control<br>(6)	1651 ± 93 (34)<br>33.2 ± 1.7	393 ± 30<br>8.6 ± 0.6	1698 ± 105 (17)<br>30.6 ± 3.0	448 ± 50<br>7.5 ± 0.8
70 ms	1675 ± 102 (32)<br>34.8 ± 2.2	379 ± 26<br>7.8 ± 0.6	1758 ± 164 (18)<br>29.6 ± 2.9	400 ± 39<br>8.9 ± 0.8
140 ms	1696 ± 109 (22)<br>31.5 ± 2.5	326 ± 28<br>6.6 ± 0.7	1836 ± 118 (22)<br>35.1 ± 3.3	**287 ± 24**<br>6.8 ± 0.8
Anova on Ranks	*0.971*<br>*0.647*	*0.358*<br>*0.214*	*0.797*<br>*0.524*	***0.028***<br>*0.095*

B **Sholl analysis: 0.5 Hz**

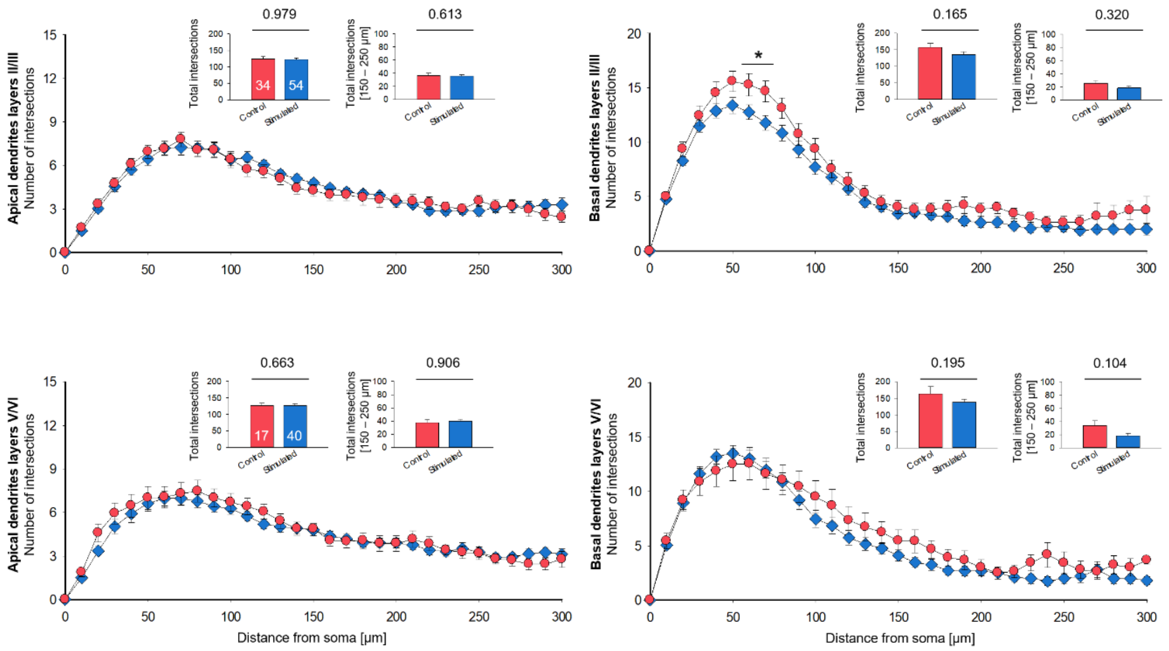

Supplementary Fig. S3. Analysis of DIV 15 cells stimulated with 2.5 Hz.

(A) *Table reports apical dendritic length (ADL) and basal dendritic length (BDL; average/cell), and the number of dendritic segments as mean ± s.e.m. for every set of neurons, as well as the number of cells/group and the number of independent batches. In bold, the statistical difference. P value determined with Kruskal-Wallis tests (ANOVA on ranks) versus handling control.* ***(B)*** *Sholl analysis of apical and basal dendrites of supragranular and infragranular pyramidal cells. Handling control in red, 70 ms and 140 ms groups were pooled and shown in blue. P values determined with Mann-Whitney ranks sum tests are given above the bars. *, p<0.05 in two bins.*
